# Research progress on the biological regulatory mechanisms of selenium on skeletal muscle in broilers

**DOI:** 10.1016/j.psj.2024.103646

**Published:** 2024-03-12

**Authors:** Shengchen Wang, Bing Tian, Yun Hu, Tingting Li, Xiaoyan Cui, Liyang Zhang, Xugang Luo

**Affiliations:** ⁎ Poultry Mineral Nutrition Laboratory, College of Animal Science and Technology, Yangzhou University, Yangzhou 225000, China; † Mineral Nutrition Research Division, State Key Laboratory of Animal Nutrition, Institute of Animal Science, Chinese Academy of Agricultural Sciences, Beijing 100193, China

**Keywords:** selenium, broiler, nutrition, skeletal muscle, antioxidant function

## Abstract

As one of the indispensable trace elements for both humans and animals, selenium widely participates in multiple physiological processes and facilitates strong anti-inflammatory, antioxidant, and immune enhancing abilities. The biological functions of selenium are primarily driven by its presence in selenoproteins as a form of selenocysteine. Broilers are highly sensitive to selenium intake. Recent reports have demonstrated that selenium deficiency can adversely affect the quality of skeletal muscles and the economic value of broilers; the regulatory roles of several key selenoproteins (e.g., GPX1, GPX4, TXNRD1, TXNRD3, SelK, SelT, and SelW) have been identified. Starting from the selenium metabolism and its biological utilization in the skeletal muscle, the effect of the selenium antioxidant function on broiler meat quality is discussed in detail. The progress of research into the prevention of skeletal muscle injury by selenium and selenoproteins is also summarized. The findings emphasize the necessity of *in vivo* and *in vitro* research, and certain mechanism problems are identified, which aids their further examination. This mini-review will be helpful to provide a theoretical basis for the further study of regulatory mechanisms of selenium nutrition in edible poultry.

## INTRODUCTION

Selenium (**Se**) is a nonmetallic element from Group 16 that was discovered in the early 18th century and derived its name from the Greek goddess of the moon named "Selene" (Rayman, 2012). Initially, selenium was regarded as a toxic element because of its ability to cause diseases in livestock. However, subsequent studies have shown that at appropriate levels, selenium can participate extensively in numerous physiological processes of mammals and many other life forms, exhibiting specific biological functions (Rayman, 2020). Until 1973, the United Nations World Health Organization (**WHO**) officially recognized selenium as an essential micronutrient for life (Foster and Sumar, 1997). Dietary selenium can be found in inorganic forms (e.g., selenite (SeO_3_^2−^) and selenate (SeO_4_^2−^) salts), organic forms (e.g., selenomethionine (**SeMet**), selenocysteine (**Sec**), and selenium-enriched yeast), and nano-selenium. The absorption, transport, and metabolism of selenium differ depending on the existing form. Once the selenium source is ingested through the diet, it will be absorbed by the intestinal tract, transported via the blood circulation to the liver for metabolism, and ultimately carried and distributed throughout the bodily tissues (Labunskyy et al., 2014). Selenium compounds from various sources are primarily metabolized into Sec, the 21st amino acid, and the main structural component of the selenoprotein family (Johansson et al., 2005; Kohrle, 2021). The diverse biological functions of selenoproteins facilitate the critical role selenium plays in maintaining cellular homeostasis and organismal health. Up to now, 25 selenoproteins have been identified in humans, with 21 of which are also being present in other vertebrates (Reeves and Hoffmann, 2009; Mariotti et al., 2012). Although excessive selenium intake is harmful to organisms, poor selenium status can lead to a range of characteristic diseases. Therefore, it is essential to maintain adequate selenium levels to ensure appropriate selenoprotein expression during various physiological and pathological processes (Delesalle et al., 2017; Zheng et al., 2019; Helke et al., 2020; Tsuji and Hatfield, 2022).

In the livestock and poultry industry, broiler chickens constitute an important type of food animal, and occupy a substantial proportion in the global meat industry, particularly in countries such as China. The health of the skeletal muscle tissue in broilers directly determines their growth progress and meat quality, which in turn affects the economic benefits gained from poultry farming. It is generally recognized that the benefits of selenium supplementation in broiler diets include increasing the selenium concentration in skeletal muscle and improving the quality of the final product; however, the precise molecular mechanisms underlying the key role selenium plays in these processes remain to be systematically elucidated. For this reason, the paper focuses on the process from selenium intake to its utilization in skeletal muscle tissue, and compares the antioxidant function of different forms of selenium compounds and their effects on broiler meat quality. Further, the molecular mechanisms underlying the regulation of skeletal muscle quality by selenium and selenoproteins are discussed to indicate an entry point for future selenium-based biological nutrition researches.

## SELENIUM METABOLISM AND SELENIUM UTILIZATION IN THE SKELETAL MUSCLE

**Figure 1:**
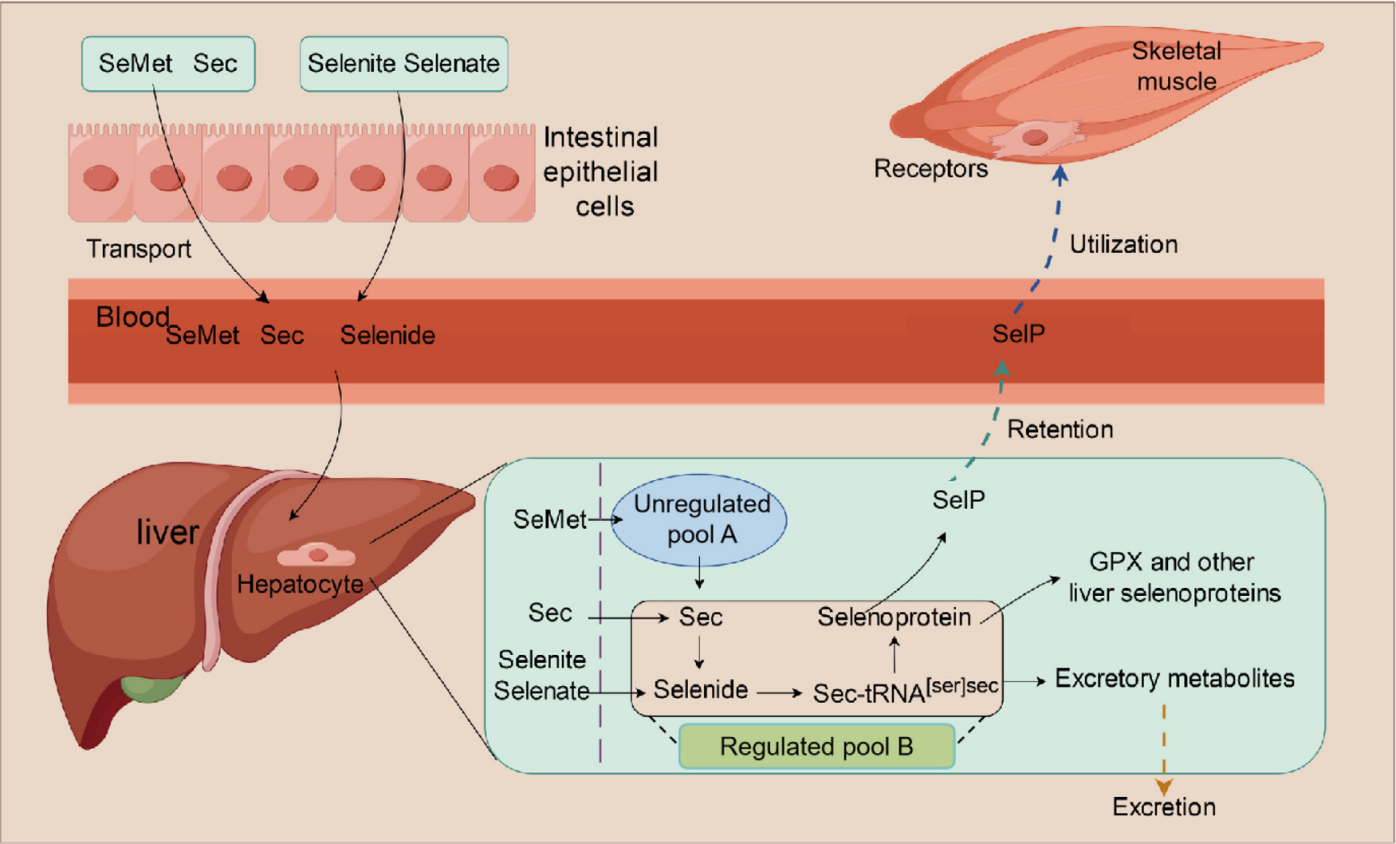
The mechanism of selenium metabolism from the intestine to skeletal muscle. SeMet = selenomethionine; Sec = selenocysteine; GPX = glutathione peroxidase; SelP = Selenoprotein P.

The dietary requirement of selenium refers to the recommended intake of this element by an organism to maintain normal physiological functions, prevent diseases, and promote overall health (Thomson, 2004; Fairweather-Tait et al., 2011). This standard was established based on scientific research and guidelines provided by professional healthcare organizations. To achieve the optimal selenium status in the body, it is necessary to ensure sufficient dietary intake, transport, and accumulation of selenium in various tissues. Currently, a comparatively systematic explanation for the mechanisms underlying selenium absorption and transport *in vivo* has been provided (Figure 1). After its absorption from the intestine into the bloodstream through the transport system, selenium from various sources initially reaches the liver, the central organ of selenium regulation. In the liver, selenium is metabolized and the excretory forms of selenium are produced to regulate whole-body selenium levels. The liver contains both the stable unregulated selenium pool A and the dynamic regulated selenium pool B (Janghorbani et al., 1990). Selenium pool A exclusively contains SeMet, whereas selenium pool B comprises all other forms of selenium compounds except for SeMet, such as Sec, Selenide, Sec-tRNA^[Ser]Sec^, and selenoproteins (Burk and Hill, 2015). In the presence of adequate selenium levels in the body, SeMet in selenium pool A tends to undergo metabolic change to facilitate storage via the methionine pathway. In time of selenium deficiency, SeMet is hydrolyzed through the transsulfuration pathway into free Sec, which then enters the dynamic selenium pool B and participates in metabolic reactions. Selenoprotein P (SelP) is the most abundant selenoprotein in plasma; it contains 10 Sec residues per molecule and plays a crucial role in selenium transport (Burk and Hill, 1994; Hill et al., 2012). After distributing selenium between retention and excretion, the liver provides reserved selenium to other tissues by secreting SelP into the systemic circulation (Burk and Hill, 2005; Richardson, 2005; Schomburg, 2022). Therefore, different tissue distributions of selenium may occur as a result of different levels of SelP uptake. Typically, upon reaching the recommended selenium level, the concentration of selenium in different organs and tissues of the body follows the order of liver > muscle > plasma (Wang et al., 2022a). Skeletal muscle tissue serves as the main selenium storage organ, accounting for approximately 50% of the total selenium content (Burk and Hill, 2015). It has been reported that low-density lipoprotein receptor-related protein 1 (**LRP1**) contributes to selenium supply by mediating SelP uptake in mice skeletal muscle (Misu et al., 2017; Takamura, 2020). However, it has not been reported whether LRP1 can also uptake SelP to maintain selenium utilization in the skeletal muscles of chickens.

The National Research Council of the United States (NRC, 1994) suggested a dietary selenium requirement of 0.15 mg/kg for broiler chickens at different growth stages. Subsequently, Feeding Standard of Chicken (2004) recommended a dietary selenium requirement of 0.3 mg/kg for broilers aged 1 to 42 d. Zhao et al. (2017) demonstrated that the skeletal muscle is also an extremely crucial selenium storage organ in broilers; chicks receiving a diet containing sodium selenite (Na_2_SeO_3_, 0.2 mg Se/kg) showed a notable increase in selenium content in the skeletal muscle tissue at 21 and 42 d of age, compared to those receiving a basal diet. Meanwhile, many studies have compared the use of different selenium sources as feed additives, concluding that organic selenium is more active, less toxic, and more bioavailable than inorganic selenium salts (Couloigner et al., 2015; Zhao, et al., 2017; Wang et al., 2021). Consequently, organic selenium offers more advantages in promoting the accumulation of selenium in broiler skeletal muscle.

## SELENIUM REGULATES SKELETAL MUSCLE ANTIOXIDANT ACTIVITY AND IMPROVES MEAT QUALITY

**Figure 2:**
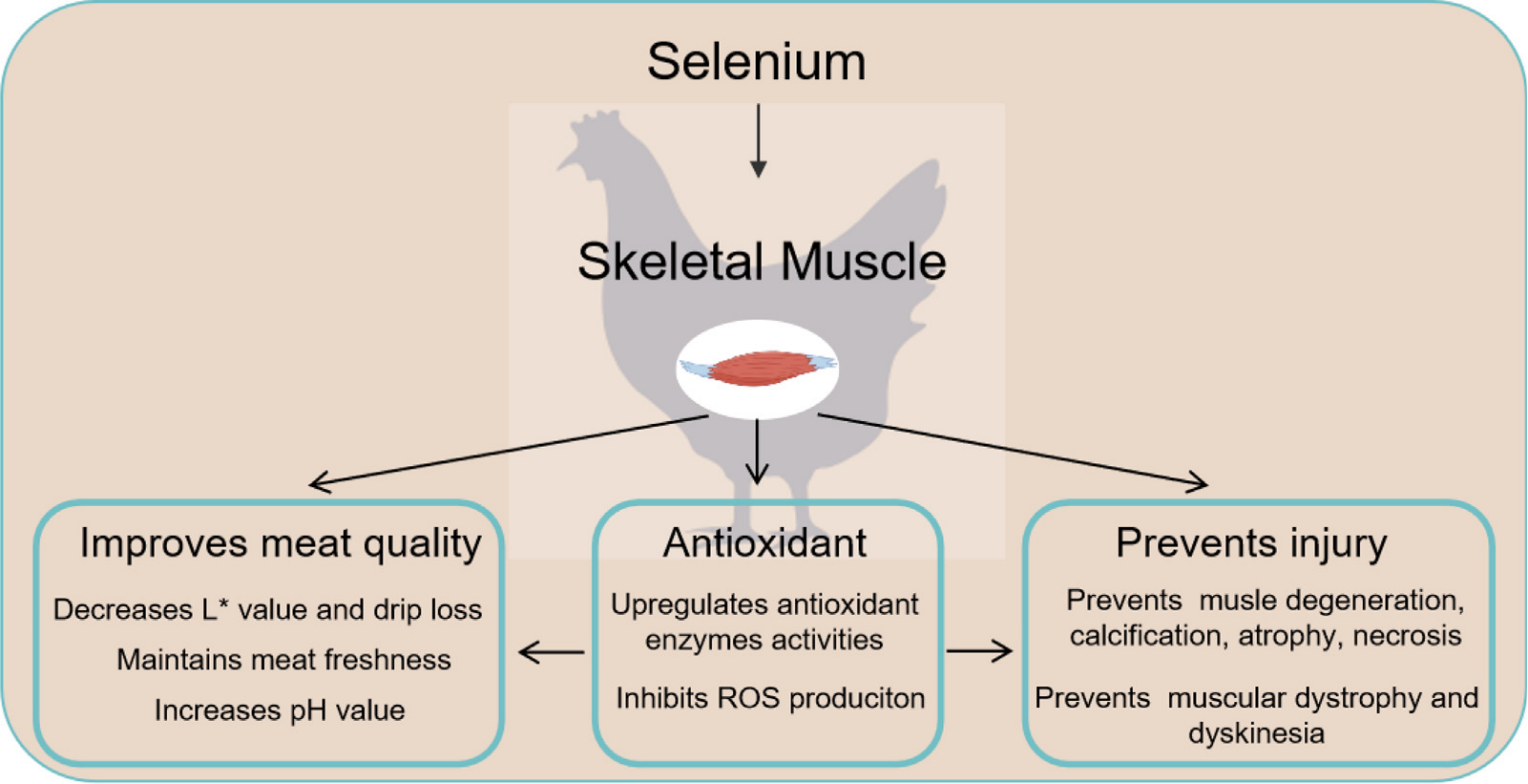
The functions of Selenium in skeletal muscle of broiler. ROS = reactive oxygen species.

In the process of raising and slaughtering livestock and poultry such as broilers, the failure of endogenous antioxidant systems and biochemical changes in muscles are considered to be among the main reasons of physiological changes and the deterioration of meat quality (Gu and Gao, 2022). Oxidative stress refers to the collapse of the antioxidant system, which commonly occurs when the production of reactive oxygen species (**ROS**) in tissue cells exceeds the scavenging capacity of the antioxidant system or when the activities of antioxidant enzymes are inhibited (Wang et al., 2022b). The resulting imbalance in the redox state leads to lipid peroxidation, which disrupts the integrity of cell membranes as well as the pigment reduction system; this leads to decreases in muscle water holding capacity, discoloration, and flavor, thereby negatively affecting the nutritional value of meat proteins (Zhang et al., 2013). Extensive researches confirmed that selenium sources and levels can help to improve the overall antioxidant capacity of tissues, thereby improving the meat quality of livestock and poultry (Grossi et al., 2021) (Figure 2). Previous studies on broiler breast muscle tissue have shown that the addition of inorganic selenium (Na_2_SeO_3_) to the diet could improve the activities of antioxidant enzymes and reduce the L* value of meat color in arbor acres (**AA**) broilers (Yao et al., 2016; Wang, 2020). Jiang et al. (2009) reported that supplementation with organic selenium in the form of SeMet in a corn-soybean meal basal diet significantly enhanced the antioxidant levels in the breast muscle of yellow feathered broilers; this enhancement was accompanied by an increase of meat pH levels and a decrease of drip loss. Meanwhile, comparative studies on chicken breast or thigh muscle tissues in broiler fed diets with inorganic selenium (Na_2_SeO_3_), organic selenium (selenium-enriched yeast, selenium-enriched plants or SeMet), and nano-selenium showed that both organic selenium and nano-selenium more effectively enhanced muscle resistance to oxidative stress, delayed meat oxidation, reduced lipid levels, and maintained meat freshness than inorganic selenium (Bakhshalinejad et al., 2019; Ibrahim et al., 2019; Hou et al., 2020; Zhao et al., 2022). In addition, numerous studies have demonstrated that the antioxidant protective effect of inorganic or organic selenium on broiler muscle tissues is equally effective in mitigating the negative impact on meat quality of various adverse factors, such as abnormal environmental temperatures and pre-slaughter transportation (Khan et al., 2018; Xu et al., 2022).

The antioxidant function of selenium mainly depends on the catalytically active sites within selenoprotein families. A total of 24 selenoproteins are present in chickens, namely selenophosphate Synthase 2 (**SPS2**), selenoprotein F (**SelF**), SelH, SelI, SelK, SelM, SelN, SelO, SelP, SelPb, SelX1, SelS, SelT, SelU, SelW, glutathione peroxidase 1-4 (**GPX1-4**), deiodinase 1-3 (**DIO1-3**), and thioredoxin reductase 1 and 3 (**TXNRD1** and **TXNRD3**) (Mariotti et al., 2012). These selenoproteins are located in different intracellular or extracellular compartments and possess distinct substrate specificities. They are highly sensitive to selenium intake and are tightly regulated by it (Huang et al., 2011; Yao et al., 2014). For instance, Hou et al. (2020) found that Na_2_SeO_3_ supplementation of diets upregulated the expressions of GPX1, GPX4, TXNRD1, and TXNRD3 in broiler breast muscle, accompanied with the increase of GPX and TXNRD activities, as well as the decrease of both drip loss and malondialdehyde (**MDA**) content. In fact, besides the widely recognized selenoproteins primarily known for their antioxidant functions (such as GPX and TXNRD), nearly all selenoproteins are also antioxidant enzymes; they have the capability to modulate antioxidant levels within tissues and cells, influence signal transduction pathways, and safeguard against oxidative damage (Schwarz et al., 2020) (Table 1). In chickens, selenoproteins have similar functions as those in humans and mice, and antioxidative selenoproteins demonstrate a high expression rate in broiler chicken muscles (Yao et al., 2014). Yao et al. (2013, 2014) reported that GPX3, GPX4, and SelW were highly expressed in broiler muscles, and SelW possesses an antioxidant function similar to the mammalian homologues, even though it lacks residue 37 (Cys37), which, in mammals is considered essential for antioxidant function. Fan et al. (2016, 2018) demonstrated that silencing SelK could regulate the expressions of other selenoproteins and increase the levels of ROS in broiler myoblasts. Given the crucial role of selenoproteins in selenium's biological functions, it is reasonable to hypothesize that they may modulate the meat quality of broiler chickens. However, further research is warranted to substantiate this assumption.

**Table 1:** The functions and mechanisms of key selenoproteins in skeletal muscle.

Selenoproteins	Functions	Mechanisms
GPX1	Improves meat quality Prevents nutritional muscular dystrophy Promotes myoblast proliferation and differentiation	Inhibits oxidative stress and apoptosis, Regulates fatty-Acid metabolism, Corrects insulin resistance
GPX4	Improves meat quality Prevents nutritional muscular dystrophy Preserves excitation-contraction coupling function Promotes myoblast proliferation and differentiation	Inhibits oxidative stress, apoptosis and ferroptosis Maintains intracellular Ca^2+^ homeostasis Regulates fatty-Acid metabolism
TXNRD1	Improves meat quality Prevents nutritional muscular dystrophy Promotes myoblast proliferation and differentiation	Inhibits oxidative stress, apoptosis and DNA injury Regulates fatty-Acid metabolism
TXNRD3	Improves meat quality Prevents nutritional muscular dystrophy	Inhibits oxidative stress, apoptosis Regulates fatty-Acid metabolism
SelK	Promotes myoblast proliferation and differentiation	Inhibits oxidative stress, endoplasmic reticulum stress, and apoptosis Maintains intracellular Ca^2+^ homeostasis
SelT	Regulates Se-deficiency skeletal muscle atrophy Promotes myoblast proliferation	Inhibits oxidative stress, and DNA injury Maintains mitochondrial homeostasis disorder Maintains oxidative phosphorylation
SelW	Prevents Se-deficiency myopathy Promotes myoblast differentiation	Inhibits inflammation reaction and oxidative stress Maintains intracellular Ca^2+^ homeostasis

## SELENIUM PREVENTS SKELETAL MUSCLE INJURY

The key reason to ensure meat quality by adequate selenium intake is to prevent skeletal muscle injury caused by oxidative stress (Chariot and Bignani, 2003; Zhao et al., 2023). The skeletal muscle is one of the main target organs for tissue damage resulting from selenium deficiency. A substantial amount of evidence supports a strong association between selenium and skeletal muscle injury. When oxidative stress is induced by selenium deficiency, skeletal muscle tissue may experience functional disruption because of vascular injury and bleeding, muscle degeneration and extensive calcification; ultimately, this can result in impaired muscle contraction mechanisms, muscle atrophy, and even necrosis, often accompanied by localized pain symptoms (Schubert et al., 1961; Van Vleet and Ferrans, 1976; Bartholomew et al., 1998) (Figure 2). Pathological dissection reveals that the tissue injury caused by selenium deficiency often occurs in muscle groups that are intensely exercised, including breast and thigh muscles; such injured tissue is characterized by dull color, localized graying or whitening (Irwin, 1977). Under both light and electron microscopy observations, skeletal muscle tissue under selenium deficiency exhibits fibers with hyaline degeneration (disruption and lysis of myofibrils, dilatation of the sarcoplasmic reticulum, destruction of mitochondrial membranes, as well as pyknosis and lysis of the nucleus) and granular degeneration (decreased density of the sarcoplasm, prominent mitochondrial swelling and distortion, as well as multiple foci of myofibrillar lysis) (Gries and Scott, 1972; Van Vleet and Ferrans, 1976; Bartholomew, et al., 1998). Due to species difference, animals exhibit diverse clinical manifestations under selenium deficiency. For broiler chickens and other domestic poultry, in addition to pathological manifestations, insufficient selenium intake can also lead to muscular dystrophy and dyskinesia (Huang et al., 2015; Yang et al., 2023).

Local or extensive tissue injury of skeletal muscle tissue is often accompanied by a repair process mediated by muscle satellite cells (also called myoblast in vitro). Skeletal muscle injury and regeneration represent distinct periods within a continuous pathological process. When the fibrous structure of the skeletal muscle is destroyed and the skeletal muscle undergoes degeneration and necrosis processes, immune cells such as macrophages are recruited and activated to ingest the dead cells (Tidball, 2011; Tidball, 2017; Le Moal et al., 2018). In order to rehabilitate damaged areas, the mononuclear satellite cells are rapidly activated from a quiescence state to initiate a multi-step process that involves proliferation, differentiation, and fusion into multinucleated myotubes or nascent myofibers. This process is primarily regulated by the specific expressions of myogenic regulatory factors (**MRF**), such as myogenic factor 5 (**Myf5**), myogenic differentiation protein (**MyoD**), myogenic regulatory factor 4 (**MRF4**), and myogenin (Charge and Rudnicki, 2004; Bernet et al., 2014; Zammit, 2017). Recent research has shown that selenium positively regulates cell viability and the myogenic differentiation in chicken myoblasts. Supplementation with appropriate levels of selenium could significantly increase the proliferation rate, the degree of myotube fusion, and the expression levels of MRFs in chicken myoblasts (Wu et al., 2012). Moreover, several studies have indicated that the expression of key selenoprotein family members can significantly affect various intracellular processes of myogenesis (Table 1). For instance, SelW not only contains binding sites for MyoD, which regulate its activity, but also prevents excessive autophagy, apoptosis, and necrosis of broiler myoblasts by regulating calcium homeostasis (Noh et al., 2010; Yao, 2016; Yao, et al., 2016). SelT regulates the cell cycle and proliferation of broiler myoblasts by maintaining mitochondrial homeostasis and oxidative phosphorylation (Wu, 2023). SelK knockdown induces apoptosis in broiler myoblasts via calcium dyshomeostasis-mediated endoplasmic reticulum stress (Fan et al., 2023). These significant findings provide a theoretical reference for the molecular mechanism of how selenoproteins regulate satellite cell myogenesis as well as the quality and health of skeletal muscle.

## CONCLUSIONS AND PERSPECTIVES

Based on the brief summary of the research progress provided, it can be concluded that selenium plays a crucial role in regulating the nutritional and quality aspects of broiler skeletal muscle. The supplementation with an appropriate selenium level has been shown to improve the meat quality and maintain the skeletal muscle health of broilers by enhancing their antioxidant function. However, it is important to note that excessive selenium intake may have detrimental effects on broiler health and growth performance. Further research is needed to explore the intricate mechanisms through which selenium and selenoproteins regulate skeletal muscle physiological homeostasis.
